# Dioxygen and Metabolism; Dangerous Liaisons in Cardiac Function and Disease

**DOI:** 10.3389/fphys.2017.01044

**Published:** 2017-12-12

**Authors:** Aude Angelini, Xinchun Pi, Liang Xie

**Affiliations:** Department of Medicine-Athero and Lipo, Cardiovascular Research Institute, Baylor College of Medicine, Houston, TX, United States

**Keywords:** cardiac metabolism, cardiac function, heart failure, oxidative stress, hypoxia, fatty acids β-oxidation, glycolysis

## Abstract

The heart must consume a significant amount of energy to sustain its contractile activity. Although the fuel demands are huge, the stock remains very low. Thus, in order to supply its daily needs, the heart must have amazing adaptive abilities, which are dependent on dioxygen availability. However, in myriad cardiovascular diseases, “fuel” depletion and hypoxia are common features, leading cardiomyocytes to favor low-dioxygen-consuming glycolysis rather than oxidation of fatty acids. This metabolic switch makes it challenging to distinguish causes from consequences in cardiac pathologies. Finally, despite the progress achieved in the past few decades, medical treatments have not improved substantially, either. In such a situation, it seems clear that much remains to be learned about cardiac diseases. Therefore, in this review, we will discuss how reconciling dioxygen availability and cardiac metabolic adaptations may contribute to develop full and innovative strategies from bench to bedside.

## Introduction

In eukaryotes, dioxygen and metabolism are intrinsically bound to energy production. And the major issue of cell metabolism is to supply the tricarboxylic cycle (TCA cycle, also called Krebs cycle or citrate cycle) in acetyl-CoA molecules and to reduce the oxido-reduction coenzymes (FADH2, NADH,H+), in order to continuously sustain the production of ATP by the mitochondrial respiratory chain. In the presence of dioxygen, cell respiration is thus hugely preferred, due to their more positive ATP production efficiency. However, any disturbance, which targets these metabolic pathways, could make the cell metabolism switch into anaerobic process, disrupting the equilibrium between oxidation/reduction processes. During the last decade, modification of both redox state and metabolism imbalance were found to be crucial factors in various physiological pathways from development to aging, and pathological processes including cancer, diabetes mellitus, neurological disorders and cardiovascular diseases (Douglas, 1963; Cunnane et al., 2011; Ma and Li, 2015).

Cardiovascular diseases represent the leading cause of mortality worldwide, while their morbidity is considered as an important economic burden for patients and for their society (Braunwald, 2013, p. 1). Heart failure is a clinical syndrome characterized by the low contractile ability of the myocardium. In the United States, more than 5 million people suffer from heart failure (HF) (Roger et al., 2012). In addition, in spite of significant declines in mortality, the 5-year survival rate is still 50% worse than cancers (Levy et al., 2002; Askoxylakis et al., 2010). Despite advances in our understanding and knowledge of HF, the complexity of the pathophysiology remains a barrier to effective therapeutic strategies. Although there are numerous etiologies that can make a heart fail, alterations of cardiac metabolism and redox-status can be depicted together as a fatal cocktail, dramatically propelling the disease to its lethal outcome (Tsutsui et al., 2011; Wang et al., 2014).

The heart is particularly sensitive to a wide range of environmental parameters, such as glucose and fatty acids (FA) blood levels or blood pressure, which are directly influenced by paracrine, endocrine hormones or neural regulators (Gordan et al., 2015). Hence, any disturbance will be able to affect its function. As a high consumer of energy, the heart must adapt its contractile activity and metabolic function to fit with the environmental availability of fuel sources and dioxygen. However, such a liability has its pros and cons. In this review, we will examine the cardiac metabolism and its involvement in cardiac disease and, conversely, how the metabolic switch from fatty acid oxidation to glycolysis and the alteration of dioxygen availability take part in the pathological progression of HF.

## Cardiac metabolism

Beating 100,000 times every day to pump about 7,500 l of blood, the heart must consume a large amount of energy in order to sustain critical and constant contraction (Abozguia et al., 2009). Although the energy demand is huge, the cardiac stock of fuel is very low, (5 μg of ATP and 8 μg of phosphocreatine per gram of tissue) sufficient for only 10 beats (Beer et al., 2002). Consequently, ATP production must be at a high rate all the time. In the adult human heart, ATP production is estimated at 6 kg per day, representing more than 20–30 times its own mass (Opie, 1968, 1969, 2004; Abozguia et al., 2009; Lopaschuk et al., 2010), demonstrating the enormous capacity of cardiac metabolism.

Under physiological conditions, cardiac metabolism involves three main steps: utilization of substrates; oxidative phosphorylation; ATP transfer and use. Changes in either of these steps may affect the myocardial energy metabolism. In the first step, cardiomyocytes take up and breakdown fuel sources. The heart is an “omnivore” organ, consuming a variety of substances to support its energy needs. A healthy adult heart mainly uses fatty acids (90%) and glucose (10%) as substrates, which are then metabolized by FA β-oxidation (FAO) and glycolysis respectively (Lopaschuk et al., 2010). FA are provided within the cells in the form of triglycerides, which are insoluble lipid complexes associated with lipoproteins or chylomicrons. Cardiomyocyte uptake of fatty acids across the plasmid membrane has been considered a passive diffusion process, but it can be usually facilitated by the fatty acid translocase CD36 (Campbell et al., 2004). Inside the cytosol, free FA are converted into fatty acyl-CoA by fatty acyl-CoA synthase. Then, the second, and most rate limiting, step is the transport of FA across the double mitochondrial membrane into the mitochondrial matrix, when the FAO occurs. For long-chain FA, a carnitine shuttle is required, facilitated by the carnitine-palmitoyl transferase 1 (CPT1) (Saggerson, 1982; Bonnefont et al., 2004). CPT1B is the major isoform located at the outer membrane of the cardiomyocyte mitochondria (Saggerson, 1982; Leij et al., 2002; Hada et al., 2012). In the heart, CPT1B represents a checkpoint that responds to metabolic feedback for FAO (He et al., 2012). Indeed, when citrate TCA intermediates are accumulated within the cells, they can be converted into MalonylCoA molecules, which represses CPT activity in a negative feedback loop, thus modulating efficiently the FAO (Saggerson, 1982; Awan and Saggerson, 1993; Reszko et al., 2004). CPT enzymes belong to the larger family of carnitine acyltransferases, which includes the carnitine O-acetyl transferases (CRATs) and the carnitine octanoyltransferases (COTs) (Hsiao et al., 2006). In this family, the carnitine binding site and the catalytic domain are particularly well-conserved. However, according to their respective ultrastructure, each subfamily would hold specific substrates preferences, these being long-chain FA for CPTs, medium-long FA for COTs and short-chain FA for CRATs (Cox et al., 1998; van der Leij et al., 2000; Hsiao et al., 2006; Seiler et al., 2014). This biochemical data could suggest that the involvement of CRATs and/or COTs in FAO is more substantial than usually thought.

Within mitochondria, fatty acyls are finally included into the β-oxidation breakdown process. FAO is a complex catabolic process comprising a sequence of four reactions: 1- Dehydrogenation, 2- Hydration, 3- Oxidation, 4- Thiolysis. Each sequence leads to the cleavage of a *n* carbons fatty acyl-CoA into an acetyl-CoA and a (*n*-2) carbons fatty acyl-CoA. Meanwhile the steps 1 and 3 require the reduction of FAD and NAD+ coenzymes respectively. Hence, finally, in theory, for a 2*n* carbons fatty acyl, it can be produced *n* acetyl-CoA, consuming *n* H_2_O and reducing back *n-1* FADH_2_ and NADH,H+ coenzymes which will lead to ATP production (Lopaschuk et al., 2010).

**Figure 1:**
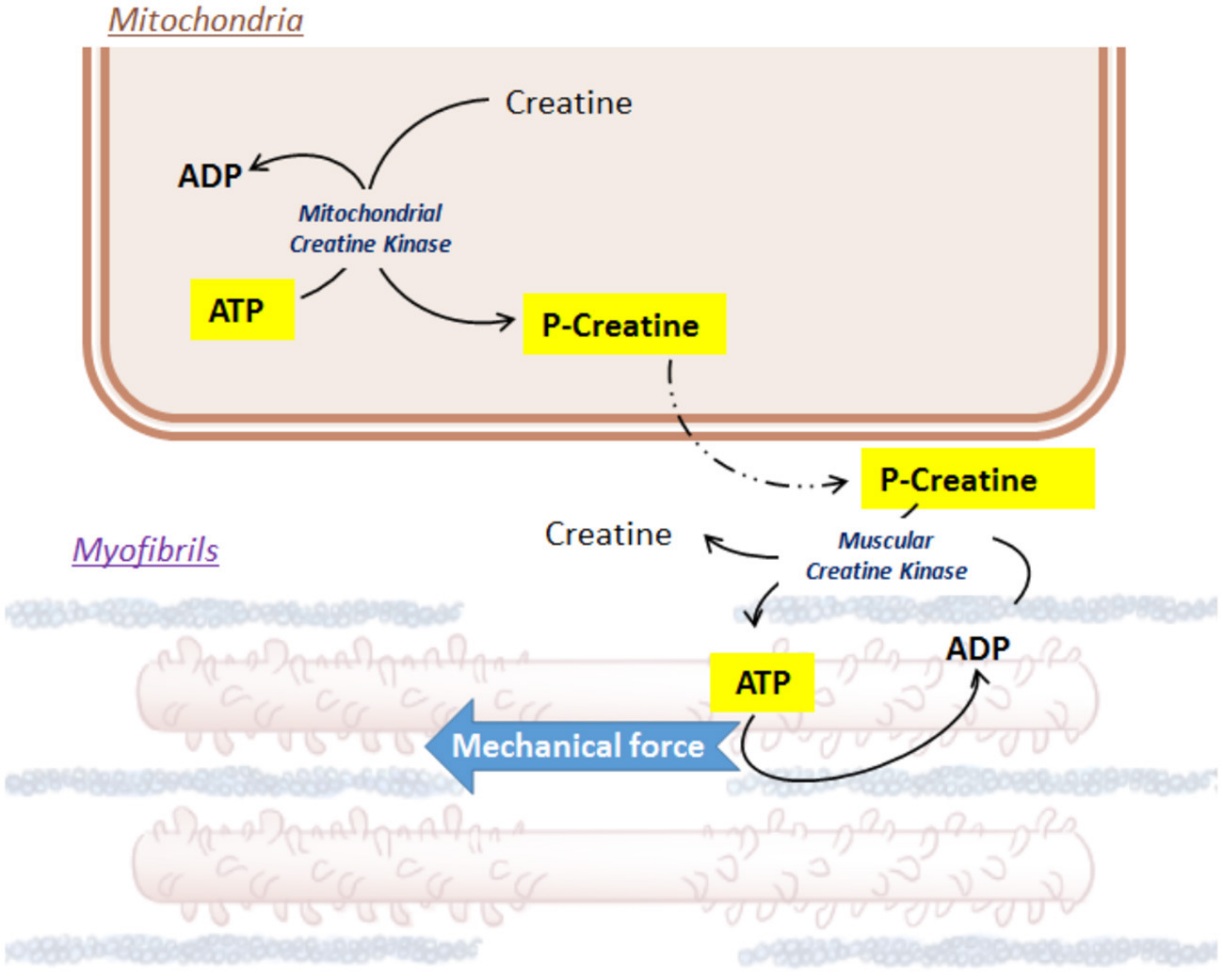
Focus on Creatine/ATP shuttle.

Notwithstanding the efficiency of FAO, glycolysis forms an integral part in cardiac metabolism. Glycolysis is able to supply in coenzymes for the TCA cycle in a less-oxygen dependent way, which preserves an equilibrium with the high-oxygen consumer FAO. In addition, beyond to the net metabolic imbalance, glycolysis intermediates can also initiate the production of the indispensable pentoses (riboses and desoxyriboses) within the cardiac cells (Wisneski et al., 1985; Barcia-Vieitez and Ramos-Martínez, 2014). Glucose cell uptake involves specific glucose transporters (GLUT), located at the plasma membrane. In cardiac muscle, GLUT1 and GLUT4 are the most represented transporters and GLUT4 endocytosis depends on insulin (Watson and Pessin, 2001, p. 4; Abel, 2004; Luiken et al., 2004; Aerni-Flessner et al., 2012). Glycolysis is a complex enzymatic process involving cytosolic kinases, isomerases and dehydrogenases (Opie, 2004). Finally, from each molecule of glucose, 2 pyruvates, 2 ATP and 2 NADH,H+ can be produced. Then, pyruvate can cross the double mitochondrial membrane, driven by specific carriers (mitochondrial pyruvate carriers, MPC1 and MPC2 (Bricker et al., 2012). On site within the matrix, pyruvate conversion into acetyl-coA is an oxidative step, which can be catalyzed by the pyruvate dehydrogenase (PDH) (Hansford and Cohen, 1978; Gray et al., 2014; Sun et al., 2015). The PDH represents another key enzyme metabolically feedback-sensitive enzyme (Stanley et al., 1996; Sugden and Holness, 2006), such that a high-amount of acetyl-CoA and NADH,H+ repress its activity, while a bigger pool of CoA and NAD+ is able to boost it (Gray et al., 2014). Finally, both glycolysis and FAO provide acetyl-CoA to fuel the TCA cycle (Barry, 2004). The TCA cycle uses acetyl-CoA as a carbon-pair donor to synthetize citrate from oxaloacetate by aldol condensation. The following steps are oxidoreduction processes, ensuring the reduction of coenzymes QH_2_ and NAD+/NADH,H+. The net ATP production is based on a proton electrochemical gradient established by the five mitochondrial respiratory chain complexes (complexes I-V), transferring an electron from NADH,H+ to oxygen. The proton uptake across the mitochondrial membrane by the F0-F1 ATP synthase (complex V) ensures the phosphorylation of ADP to ATP. Lastly, to ensure contraction of the heart muscle cells, ATP must be brought into the proper utilization site, the muscle fibers. However, the mitochondrial double membrane is roughly permeable to this molecule. Local mitochondrial creatine kinase initiates the energy shuttle to the cytosol by catalyzing the transfer of a high-energy phosphate from ATP to creatine, releasing ADP and a high-energy phosphocreatine (Ingwall et al., 1985; Wallimann et al., 1998; Schlattner et al., 2006; Figure 1). Due to its smaller size, phosphocreatine easily diffuses from mitochondria to myofibrils, where the muscular creatine kinase converts back energy from phosphocreatine into ATP, releasing creatine (Ingwall et al., 1985; Schlattner et al., 2006; Zervou et al., 2016). In turn, this ATP is used by actin-myosin complexes inside the myofibrils and converted into mechanical force.

## Oxygen consumption and the double-edged redox signaling in cardiac cells

The heart is the highest dioxygen consumer of all organs. Globally, 8–15 mL of dioxygen are perfused per min per 100 g of resting heart, and this rate can increase up to 6–7-fold during exercise, to match closer to ATP needs (Klabunde, 2012). Almost 90% of dioxygen is burnt within the mitochondria as an electron donor for oxidative phosphorylation. However, a lesser amount of dioxygen is used by the oxidase enzymes, mainly NADPH oxidases (Bedard and Krause, 2007; Lassègue et al., 2012) xanthine oxidases (Cantu-Medellin and Kelley, 2013; Battelli et al., 2016) and monoamine oxidases in cardiac cells (Viel et al., 2008; Kaludercic et al., 2014; Vendrov et al., 2015). However, for a minor portion of dioxygen (<2%), reduction is uncompleted, leading to the generation of reactive oxygen species (ROS), and especially the most unstable superoxide anion (O_2_•−), which can react with itself to produce the non-radical hydrogen peroxide (H_2_O_2_), and then OH•− by the Fenton reaction in presence of Fe^2+^, or OH by Haber-Weiss reaction (Andreyev et al., 2005; Chen et al., 2012). In addition, Nitric oxide synthase can also use O_2_•− to product nitric oxide (NO•), the precursor of reactive nitrogen species (RNS), leading to ONOO- and ONOOCO_2_ when they reacted with O_2_•− and CO_2_/HCO_3_- respectively (Augusto et al., 2002).

Thus, ROS/RNS are a common by-product of cellular respiration and metabolism, and their cellular level is tightly regulated by antioxidant systems including enzymatic or non-enzymatic scavenging strategies (β-carotene, ascorbic acid, glutathione-SH, etc.) (Andreyev et al., 2005; Nediani et al., 2010; Chen et al., 2012). Non-exhaustively, enzymatic ROS scavenging system involves the superoxide dismutases that catalyze the O_2_•− conversion to H_2_O_2_. Then, H_2_O_2_ can be decomposed in H_2_O by glutathioneperoxidase-1 and -4 or catalases for a lesser extent. However, an other important scavenging system involves peroxiredoxin-3 (Prx3)/Thioredoxin 2 (Trx2) and Thioredoxin Reductase-2 (TrxR2) axis (Stanley et al., 2011; Cunniff et al., 2014; Li H. et al., 2017). Recent work described TrxR2 as a major controller of H_2_O_2_ in mammalian heart mitochondria (Stanley et al., 2011). Interestingly, it has also been demonstrated that energization status of respiring mitochondria directly correlates with Trx2 level, thus suggesting a crucial role of Trx2/TrxR2 and Prx3 axis in mitochondrial function and furthermore in cardiomyocyte ATP production (Stanley et al., 2011).

Hence there are numerous and sophisticated scavenging strategies within healthy cardiomyocytes, and yet ROS remain at a minimal level. In fact, under physiological context, ROS/RNS hold beneficial and protective virtues. For instance, nitric oxide (NO•) regulates endothelium-dependant epicardial and microvascular vasodilation, favoring dioxygen perfusion in response to metabolic stimulation (Quyyumi et al., 1995). Moreover, ROS/RNS are involved as upstream effectors of cell signaling pathways *via* the now well-known redox signaling (Burgoyne et al., 2007; Nediani et al., 2010). One of the most redox-sensitive pathways is PI3K/AKT that plays a central role in cardiac metabolism and insulin sensitivity (Cook et al., 2002; Sugden, 2003; Morisco et al., 2005; O'Neill et al., 2007). As upstream regulators of MAPK, ERK1/2, p38, and JNK signaling pathways and kinases such as PKC or the histone deacetylates HDAC4 class II, ROS can impact on main transcription factors activity, such as NFAT, MEF2, SRF, and GATA4, which finally orchestrates protein synthesis, cardiomyocyte survival and differentiation, especially by Sabri et al. (1998), Wei et al. (2001), Matsushima et al. (2013), Barajas-Espinosa et al. (2015). In cardiac cells, redox signaling also impacts on main steps of myocyte contractility by targeting Ca^2+^ exchangers (SERCA2 or the ryanodine receptor) (Sharov et al., 2006; Bellis et al., 2009; Nediani et al., 2010; Tang et al., 2010; Snijder et al., 2011; Simon et al., 2014). Then, ROS have a clear impact on mitochondrial permeability by promoting the opening of the ATP potassium channel and the closure of the mitochondrial permeability transition pore (MPTP) (Cho et al., 2014). Furthermore, RNS (specifically the nitroxyl, HNO donor) can modulate myofilaments Ca^2+^ sensitivity. In fact, they ensure reversible formation of disulfite bonds between cysteine residues, which improves heart muscle contractility (Gao et al., 2012). Last but not least, according to recent studies, physiological cardiomyocyte stretching leads to an increase of ROS production by NOX2 in a microtubule-dependent manner. This mechano-chemo transduction pathway, called “X-ROS,” favors a surge of Ca^2+^ release due to a “tuning” of Ryanodine Receptors (Prosser et al., 2011, 2013; Ward et al., 2014). In addition, NOX2-induced oxidation of Ca^2+^/Calmoduline-dependant kinase, which has been previously described as an intermediary Ca^2+^ signaling and redox signaling, could be also involved in X-ROS redox signaling (Erickson et al., 2008; Snijder et al., 2011; Ward et al., 2014). Consequently, ROS/RNS are able to act as safeguards of mitochondrial respiratory and metabolic activity, but they remain at the border between physiological and maladaptive cardiac remodeling processes.

Oxidative stress occurs when there is an imbalance between production and scavenging of ROS, else due to an excess of production by the main sources, and/or by a decrease of antioxidant scavenging systems. Contrary to the virtues mentioned previously, under such a context, ROS/RNS become poisonous for the cells when the cellular antioxidant defenses become overwhelmed (Djordjević et al., 2008; Csányi and Miller, 2014). Due its high amount of metabolic activity and dioxygen consumption, the heart is particularly predisposed to excess of ROS. And, since they are particularly unstable and deleterious, ROS can combine with proteins or lipids in a more unspecific and irreversible manner, which can thus disrupt membranes integrity, cytoskeletal organization, metabolic and ionic homeostasis, and enzymatic stoichiometry (Giordano, 2005; Misra et al., 2009; Tsutsui et al., 2011; Csányi and Miller, 2014). During ischemia, myofilaments oxidation by carbonylation leads to a decrease in both Ca^2+^ sensitivity and myosin Ca^2+^ ATPase activity, resulting in a decrease in sarcomere contractility (Avner et al., 2012; Balogh et al., 2014). In addition, contrary to physiological process, ROS/RNS can trigger cellular Ca^2+^ overload, which can reduce Ca^2+^ sensitivity within the muscle fibers, thus finally triggering the contractility defect and rushing the cardiac cell toward necrosis or apoptosis (Ferrari et al., 2004; Giordano, 2005). Excess of ROS may exacerbate maladaptive process (Richters et al., 2011; Penna et al., 2013). For instance, ERK1/2 and HDAC4 II activation will favor cardiomyocyte hypertrophy, while overactivation of JNK could promote apoptosis (Matsushima et al., 2013; Mutlak and Kehat, 2015; Kanaan and Harper, 2017). Oxidative stress could lead to cysteine oxidation of cGMP-dependant protein kinase (PKG) (Burgoyne et al., 2007). Recent studies demonstrates that expressing of a “redox dead” PKG1α^C42S^ relatively protects the mice from hemodynamic-stress-induced adverse effects (Prysyazhna et al., 2012; Nakamura et al., 2015). By affecting both mitochondrial proteins and phospholipids, ROS also exacerbate the disruption of the mitochondrial respiratory chain and the loss of ATP production. Additional studies described that increase of ROS production by xanthine oxidase is involved in impaired energy metabolism during heart failure and myocardial infarction, and allopurinol (inhibitor of xanthine oxidase) prevents these deleterious effects (Wang Z. et al., 2016; Schuchardt et al., 2017).

Cardiac metabolism and adaptation are thus widely dependent on redox balance within the cells of the myocardium. Meanwhile, ROS level is deeply sensitive to the energy performance within cardiomyocytes. Recently, it has been demonstrated that oxygen consumption and H_2_O_2_ production are directly proportional to level of long-chain palmitoyl-CoA concentration (Cortassa et al., 2017) which perfectly matches with this concept. This also underlines the significant issues that drive heart metabolic adaptions. Consequently, linked together, this amount of knowledge emphasizes ambiguous roles of ROS in cardiac physiology and pathology.

## Lability and physiological adaptation within the heart

When the heart is correctly perfused with adequate dioxygen concentration, up to 95% of the ATP production is due to oxidative phosphorylation of FA inside the mitochondria, while the remaining ~5% is provided by glycolysis and to a lesser extent the TCA cycle (<1%) (Scolletta and Biagioli, 2010). For 1 mole of an unsaturated long-chain fatty acids like palmitate, the net energy production is estimated at 128 ATP, whereas this rate reaches at only 38 ATP for 1 mole of glucose (Stanley et al., 1997). However, in comparison to glycolysis, FAO uses 12% more dioxygen and thus FAO is considered less dioxygen-efficient (Mjos, 1971; Grynberg and Demaison, 1996; Afanasiev et al., 2016). Consequently, even a major component of cardiac energy production, FAO is thus particularly sensitive to dioxygen concentration, and a tight equilibrium must be kept between availability of fuel sources. Dioxygen must be available during ATP production, and ATP is consumed during contraction. Therefore, the heart requires some flexibility and adaptability, metabolically switching between FAO and glycolysis when needed for optimal functionality. For almost five decades now, it has been well established that a reciprocal imbalance links FAO and glycolysis. This competitive process, historically described as the “Randle cycle” (Neely et al., 1972; Randle, 1998), is such that an increase of availability for one fuel source will favor its use, repressing the use of the others. For instance, an excess in acetyl-CoA production from FA breakdown inhibits PDH, thus reducing the glycolytic rate (Stanley et al., 1996; Sharma et al., 2005). By contrast, an activation of glycolysis favors the activity of acetyl-CoA carboxylase, which in turns increases the level of malonyl-CoA, thus reducing CPT1-dependant FA shuttle and β-oxidation (Awan and Saggerson, 1993; Reszko et al., 2004).

However, additional regulatory processes participate in this cardiac metabolic flexibility, beyond whatever substrate is available. These processes involve a wide range of biomolecular and biochemical parameters including protein activity and translocation as well as allosteric modulation of enzymes but also gene expression (nuclear and mitochondrial gene program). Among them, PPARs and PGC-1α represent powerful regulators of cardiac metabolism (Barger and Kelly, 2000; Campbell et al., 2002; Duncan and Finck, 2008; Finck et al., 2008; Rowe et al., 2010). PPAR isoforms (α, β, δ) can bind to Retinoid X Receptor (RXR) transcriptional factor, whereas PGC-1α secondly acts as a co-activator, increasing the transcriptional activity of the RXR-PPAR heterocomplex. PPARα is the major isoform in the cardiac muscle, whereas PGC-1α is more ubiquitous but particularly sensitive to a wide number of extracellular or physiological stimuli such as starvation and exercise in the striated muscles (Lopaschuk and Spafford, 1990; Miura et al., 2008; Chinsomboon et al., 2009). In intracellular compartment, PGC-1α is also a downstream effector of AMPK as well as cAMP and calcineurin signaling pathway (Schaeffer et al., 2004; Jäger et al., 2007; Miura et al., 2008; Chinsomboon et al., 2009). PPARα/RXR/PGC-1α heterocomplex is mostly dedicated to FA activation, transport and breakdown, through the expression of fatty acyl-CoA synthase, CPT1B, and long-chain fatty acyl-CoA dehydrogenase, but also PDH (Barger and Kelly, 2000; Hopkins et al., 2003; Duncan and Finck, 2008). By contrast, targets of PPAR β/δ are involved in both FAO (CPT1B and malonyl-CoA decarboxylase) and Glycolysis (activating especially GLUT4 receptor and Phosphofructokinase) (Burkart et al., 2007; Risérus et al., 2008).

**Figure 2:**
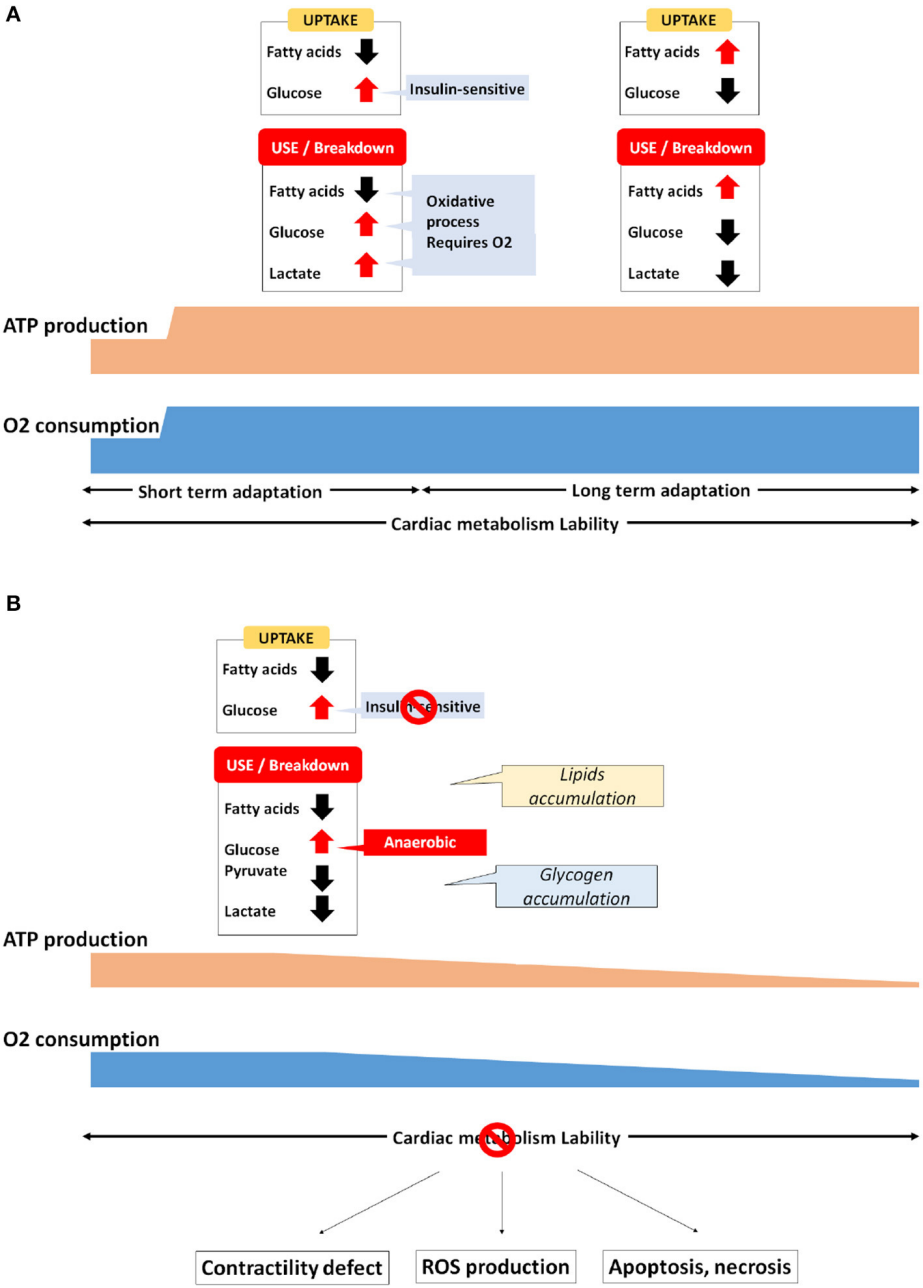
Metabolic adaptation in the cardiac muscle **(A)** Physiological, **(B)** Pathological.

Also contributing to the metabolic homeostatis, endocrine hormones (insulin or glucagon-like peptides) and neurohormones (catecholamines) can adapt rapidly several parameters such as blood FA or glucose levels, heartbeat or blood pressure, thus connecting metabolic adaptation of the heart to the whole organism and reciprocally (Gordan et al., 2015). Whereas during normal homeostasis the maintenance of cardiac metabolism is relatively subtle, the heart's energy needs can evolve suddenly and drastically. For example, athletic exercise can increase cardiac output up to 6-fold from resting values, thus requiring a proportional increase in ATP production (Warburton et al., 2002). Under such a context, cardiac metabolism reaches peak activity (Figure 2A). Increases in myocardial workload typically boost FA uptake and FAO. But the glycolysis is also stimulated by exercise in human, or by other stressors such as β-adrenergic or workload stimulation in the hearts of both large animals and rats (Andersson et al., 1991). In addition, exercise increases lactate blood level, which leads to elevated lactate use and oxidation by the cardiac muscle (Kaijser and Berglund, 1992; Jeffrey et al., 1995; Goodwin and Taegtmeyer, 2000), notably by decreasing fatty acids uptake and oxidation (Schönekess, 1997). By contrast, during prolonged exercise of more than 30 min, fatty acids are released by the adipose tissue in the blood circulation, favoring their absorption by the heart (Martin et al., 1993). Meanwhile, it has been demonstrated that the level of malonyl-CoA decreases after 15–30 min of high pacing-induced stimulation in the hearts of pigs and rats, thus facilitating the removal of CPT1 inhibition (Goodwin et al., 1998; Winder, 1998; Zhou et al., 2008). According to this data, longer-term exercise tolerance seems to be associated with the myocardial re-uptake and breakdown of FA. However, an abrupt increase of workload in pigs leads also to a 2.5-fold increase of FAO, although the level of both malonyl-CoA or AMPK remains unchanged (King et al., 2005). Seemingly contradictory, these studies suggest that acute adaptation involves different pathways that remain poorly investigated.

## Metabolic adaptations, beyond good and evil in cardiac diseases

In cardiovascular diseases, regardless the etiology, energetic starvation is described as a fairly common feature. However, specific metabolic alterations can vary widely from one patient to another (Neubauer, 2007; Turer et al., 2010). This illustrates how the whole complexity of cardiac metabolism under pathological context remains fairly comprehended.

In hypertrophic remodeling, increases in hemodynamic pressure and workload urge the heart to reinforce its contractile strength by increasing its muscular mass. Contrary to most somatic cell types, cardiomyocytes are no longer able to proliferate (Ahuja et al., 2007; Zebrowski and Engel, 2013). Hence, they must increase their size and their stock of both sarcomeres and mitochondria to sustain the upgraded myocardial needs. Cardiomyocyte hypertrophy also requires some metabolic adaptation. Contrary to physiological remodeling, pathological hypertrophy is characterized by a return to fetal gene program that favors anaerobic glycolysis over that of FA, pyruvate and lactate oxidative breakdown (Christe and Rodgers, 1994; Sack and Kelly, 1998; Barger and Kelly, 1999; van der Vusse et al., 2000; Lehman and Kelly, 2002a,b; Ingwall, 2009). In addition, data in both rat and human has validated the switch toward anaerobic glycolysis in pathological cardiac hypertrophy (Ritchie and Delbridge, 2006; Ma and Li, 2015). In hypertrophied hearts, the level of PGC-1α and PPAR is also decreased (Barger and Kelly, 2000; Duncan and Finck, 2008). Conversely, the same maladaptive fetal metabolic patterns are observed in the heart of transgenic mice lacking PPARα, and these are associated with a significant increase in malonyl-CoA (Campbell et al., 2002; Finck et al., 2008). Moreover, an overexpression of PPARα in neonatal rat cardiomyocytes is able to counteract the hypertrophic remodeling process, which suppresses protein synthesis and mitigates CPT1 reduction Cardiac-specific disruption of PPARβ/δ is also associated with cardiac hypertrophy development and a decrease of FA oxidation (Cheng et al., 2004). Above-mentioned activation of PPAR/PGC-1α pathway is sufficient to prevent the decline of FAO, thus maintaining FA oxidation rate. Moreover, increased cardiomyocyte size is associated with a decrease in oxygen tension per area, stabilizing the hypoxia-inducible factor 1α (HIF1α) (Des Tombe et al., 2002; Chu et al., 2012; He et al., 2016). HIF1α takes part in the subtle readjustment of anaerobic glycolysis by favoring fetal lactate dehydrogenase (LDH-A) and pyruvate dehydrogenase kinase (PDK1) activity along with hypoxia-induced GLUT1 expression (Brahimi-Horn et al., 2007). Initially, the fact that GLUT1 expression now supersedes GLUT4 favors insulin resistance. Then, despite glucose uptake being particularly enhanced, pyruvate and lactate oxidation remains low, because their activity is drastically repressed by the increase of PDK1 and the replacement by LDH-A, respectively. In addition, it also well known that hypoxia is able to repress FA metabolism gene expression (Brahimi-Horn et al., 2007). This further validates the relationship between HIF1α, PPAR expression, and cardiac metabolism, thus accordingly reinforcing the notion of the Randle cycle switch in heart failure scenarios (Figure 2B).

Pathological hypertrophy can progress quickly toward HF. Although etiology of HF is particularly complex, metabolic disruption is clearly involved in this progression (Ingwall and Weiss, 2004; Doenst et al., 2013; Abdurrachim et al., 2015). In mild-to-moderate HF, it is generally thought that phosphocreatine level decreases whereas upstream cardiac metabolism toggles from FAO toward glucose/pyruvate oxidation (Sack et al., 1996; Ye et al., 2001; Phillips et al., 2010). As mentioned previously, phosphocreatine is the substrate of creatine kinases that actively take part in ATP buffering. Gupta et al. demonstrated that ATP flux and myocardial contractility are directly related to myocardial CK (M-CK) expression and activity (Gupta et al., 2012). In fact, while overexpression of M-CK in mouse keeps higher ATP flux and better cardiac function, especially at 4 weeks following thoracic aortic constriction; this beneficial impact is lost when M-CK is raised back to the normal level (Gupta et al., 2012). Hence the failing heart is definitively an engine “out of fuel” (Neubauer, 2007), and this is directly related to M-CK activity (Gupta et al., 2012). As long as it can be supplied with adequate fuel, the heart is able to maintain its contractile activity, albeit at a lower output. However in end-stage HF, oxidation of FA, pyruvate and lactate is shunted, favoring the lower dioxygen-consuming glycolytic pathway (Ussher, 2014). In human idiopathic dilated cardiomyopathy, a common precursor to HF, FA uptake and oxidation are substantially decreased in patients (Dávila-Román et al., 2002). Furthermore, metabolic assays performed in rat and human hearts (by mass spectrometry or by ^14^C and ^3^H radioactivity measurements) strongly ascribed a correlation between FA deregulation and HF severity (Turer et al., 2009, 2010; Doenst et al., 2010). In addition, during pacing stress, the over-activation of glucose uptake, usually occurring in control groups, is disrupted in both human DCM patients and canine models of DCM (Nikolaidis et al., 2004a,b; Bergman et al., 2009). Taken together, this amount of data points out a lack of metabolic-related responsiveness as a common feature of HF.

In addition, in such a context of failing heart, dioxygen level (availability or starvation) represents another critical point in occurring maladaptive process. Consequently, signaling pathways that contribute to metabolic switching in the heart differ significantly between non-ischemic HF (when dioxygen remains perfused) and ischemic HF (when heart becomes dioxygen-straved).

Metabolic disturbances, in non-ischemic HF, remain close to those leading to HF, such as the switch from FA to glucose utilization. For instance, in an experiment on pressure overload, using mice, transverse aortic constriction (TAC) is followed by an acute increase of myocardial glucose use in the day following the experiment, and this rate reached at higher level 7 days after TAC (Zhong et al., 2013). Additional data demonstrated that the metabolic switch is still preserved 6–8 weeks after TAC in mice (Kolwicz et al., 2012; Pereira et al., 2013, 2014), and this has been also validated in other animal models of pressure overload or volume overload or HF in rats, rabbits and dogs (Taegtmeyer and Overturf, 1988; Allard et al., 1994; Christe and Rodgers, 1994; Christian et al., 1998; Labinskyy et al., 2007). Another study, this time in patients suffering from non-ischemic DCM, right ventricle glucose uptake had been monitored by PET imaging (using the radioactive isotope [^18^F] fluodeoxyglucose) (Wang L. et al., 2016). Conclusions of these experiments demonstrated a correlation between increased right ventricle glucose uptake and dysfunction (Wang L. et al., 2016). In addition, a recent paper established the metabolomics profile of non-ischemic HF in patients (Mueller-Hennessen et al., 2017). In this clinical study, plasma metabolite profile has shown to widely differ between HF patients and controls, even during resting time, while cardiopulmonary exercise testing clearly exacerbated these differences (Mueller-Hennessen et al., 2017). More precisely, in this article, it has been demonstrated a clear decrease of complex lipids and FA, counterbalanced by an increase in glutamate and purine byproducts, whereas glycolysis was impaired (which is also established as a marker of pre-diabetes) (Mueller-Hennessen et al., 2017). However, in ischemic HF, inefficient blood supply by coronary arteries leads to oxygen starvation within the myocardium, impairing both contractile activity and metabolic imbalance. Not surprisingly, it stands to reason that ischemia compromises all the oxidative steps involved in cardiac metabolism. It reduces the ATP production from both FA and pyruvate but increases anaerobic glycolysis, in proportion with the degree of ischemia (Ferrari et al., 2004; Turer et al., 2009). In such a low oxygen context, the remaining part of oxidation always favors FAO rather than pyruvate use. By contrast, pyruvate is mostly converted into lactate, in order to fuel anaerobic glycolysis in NADH,H+ coenzyme (Josan et al., 2014; Chen et al., 2015; Mariotti et al., 2016). Consequently, to avoid a massive acidification inside the failing cardiomyocytes, an extended part of this newly-produced ATP is dedicated to ionic exchange and homeostasis. The accumulation of reducing coenzymes (NADH,H+ and FADH_2_) shunts the activity of several metabolic enzymes, such as acyl-CoA dehydrogenase or 3-hydroxylacyl-CoA dehydrogenase, which are directly sensitive to redox state (Nocito et al., 2015). Thus, FA-derived intermediate metabolites accumulate rapidly within the cardiac cells. Fatty acyl CoA favor mitochondrial storage, whereas fatty acyl-carnitines may be accumulated in both mitochondria and other cellular compartments (Kler et al., 1991; Fukushima et al., 2015). Such a build-up of fatty acyl esters and carnitine triggers disturbances in mitochondrial morphology (irregular cristae, amorphous densities) which in turn impact on metabolic function (Kuzmicic et al., 2014; Elezaby et al., 2015). In the failing heart, mitochondrial dysfunction is, indeed, a well-established common feature (Huss and Kelly, 2005; Banović and Ristić, 2016; Goh et al., 2016). At first, their morphology is usually affected, since they are described as smaller in size with membrane disruption and matrix depletion. In these morphologically altered mitochondria, the complex integrity of the respiratory chain is abrogated, ATP production is reduced, and the level of ROS dramatically increased (Banović and Ristić, 2016; Sverdlov et al., 2016). As previously mentioned, excess of ROS will lead to protein degradation, loss of membrane integrity, Ca^2+^ overload, thus propelling the cardiomyocytes to necrosis and apoptosis (Giordano, 2005; Misra et al., 2009; Sanada et al., 2011; Tsutsui et al., 2011; Csányi and Miller, 2014). While hypoxic conditions leads cardiomyocytes to drastic adaptations, during reperfusion phase following ischemia, cells suffer this time from a sudden burst of dioxygen. Under such an acute re-oxygenation context, a high and uncontrolled release of ROS occurs, due to functional remaining mitochondria and re-activated xanthine oxidase, and this reveals as particularly deleterious (Sanada et al., 2011; Raedschelders et al., 2012; Zuo et al., 2013). Myocardial infarction in mice is notably characterized by a higher level of myosin and actin carbonylation, resulting in a decrease in Ca^2+^ sensitivity and myocardial dysfunction (Dalle-Donne et al., 2001; Castro et al., 2013; Balogh et al., 2014). In addition, actin oxidation, which is NOX-dependent phenomenon, reorganizes the thin filaments, which favor cell survival in yeast (Farah et al., 2011; Wilson et al., 2016). In the case of failing cardiomyocytes, we can suggest that myofilament oxidation could prevent depolymerization and stabilize actin-myosin complex, which finally reduces ATP needs. Hence, resulting sarcomeres can sustain their contractility, even in a slower, weaker but less energy-consuming fashion. While long-term ischemia following by reperfusion is clearly a deleterious cocktail for the heart, surprisingly, acute but repetitive periods of ischemia is beneficial and cardioprotective (Eltzschig and Eckle, 2011; Frank et al., 2012; Zuo et al., 2013). In fact, this “warm up” effect (called ischemic preconditioning, IPC) is able to improve recovery after a myocardial infarction and to reduce the severity of arrhythmia (Yang et al., 2010). A wide number of research works was led in order to elucidate the mechanisms involved in this surprising phenomenon. Briefly, it has been demonstrated that IPC positively impacts on sarcolemmal and mitochondrial ATP K+ channels. This prevents deleterious ischemia-induced Ca^2+^ overload and could favor ATP production by impacting on adenylate cyclase and muscular creatine kinase (Turrell et al., 2011; Zuo et al., 2013; Donato et al., 2017). In addition, within mitochondrial matrix, influx of K+ inhibits mitochondrial respiratory chain (especially Complex II) and favors the matrix swelling, which was described as an efficient enhancer of FAO and ATP production (Kaasik et al., 2007; Zuo et al., 2013; Donato et al., 2017; Javadov et al., in press). Due to the rapid switch between oxygen starvation/reperfusion during this phenomenon, the role of ROS was rapidly suspected in IPC (Bolli et al., 1989; Qiu et al., 1997). An acute increase in ROS was indeed taken in evidence when short periods of ischemia were challenged, resulting in K+ ATP channels activation (Gross et al., 2007). Furthermore, beneficial effects of IPC are critically blunted when antioxidants are administrated, validating crucial and beneficial impact of ROS in IPC (Skyschally et al., 2003; Khanna et al., 2008). More specifically, a recent research study demonstrated that a surge of O_2_•− arises within the first minute following ischemia (Zhu and Zuo, 2013). In particular, this early burst of ROS is due to myoglobin oxidation, releasing O_2_•− (Zhu and Zuo, 2013). Myoglobin (Mb) is an oxygen- and iron-binding hemoprotein that ensures buffering dioxygen in striated muscle cells. Beyond this traditional function, myoglobin is also an oxygen-sensitive safeguard of NO homeostasis, since oxygenated Mb acts as NO scavenger in normoxia, while Mb oxidation favors NO generation during hypoxia (Rassaf et al., 2007; Totzeck et al., 2014). NO, generated from myoglobin oxidation reduces cardiac oxygen consumption and energy status, which helps to prevent myocardial damages during short phases of hypoxia or ischemia/reperfusion (Hendgen-Cotta et al., 2017). Moreover, based on its oxidation status, myoglobin can bind to FA (especially with long chain oleate and palmitate) (Götz et al., 1994; Shih et al., 2014, 2015; Jue et al., 2016). A recent work (using Mb −/− mice) demonstrated that myoglobin has a new crucial role in FAO by preventing lipid accumulation and alleviating FAO in cardiomyocytes, which preserves cardiac function (Hendgen-Cotta et al., 2017). Such an amount of data reinforces the idea that dioxygen availability and metabolism are deeply bounded in cardiac adaptation, and myoglobin oxidation seems to actively take part in this process as being a ROS/RNS/metabolic sensor in cardiomyocyte. More broadly, while ROS/RNS have been described in a Manichean viewpoint for years, ischemia/reperfusion and IPC provide evidences that they definitively work as a double-edged sword, based on other critical parameters influencing cardiomyocyte needs (especially dioxygen and fuel).

This is the reason why therapeutic strategies are still such challenging in cardiac diseases.

## Discussion: therapeutic strategies

Cardiac metabolism attracted closer attention in the last decade, appearing as a promising therapeutic target. However, despite the range of accumulated data and technical skills dedicated to assessing this topic (Taegtmeyer et al., 2016), the whole understanding suffers from numerous gaps which remain poorly explored. Meanwhile, although our knowledge of cardiac diseases is expanding, most treatments are still only moderately effective. Hence, many strategies have been envisaged for which we will discuss below the pros and cons in the view of the most recent publications.

### Antioxidant strategy

In the first instance, antioxidant agents demonstrated a therapeutic appeal (for an exhaustive review and discussion about this topic, refer to Goszcz et al., 2015). Briefly, two main strategies have been developed in the past decades: else supply in natural antioxidants, or inhibit the main deleterious sources of ROS.

Primarily, several trials have used vitamins and enzymes, due to their ROS scavenging properties. In particular, it has been demonstrated that addition of retinoic acid and catalase in the culture medium inhibits Angiotensine II/TNF-α-induced hypertrophy in neonatal rat ventricular cardiomyocytes (Nakamura et al., 1998). Following these promising results, a larger-scale clinical study was launched in order to investigate the clear impact of antioxidant treatment in HF. Called HOPE (Heart Outcomes Prevention Evaluation), this study resulted in disappointing results, taken in evidence that higher dose of retinoic acid is pro-oxidant, worsening of the cardiac defect (Sleight, 2000; Gerstein, 2001). In addition, some studies trend to suggest vitamin E and vitamin C can be cardioprotective in patients, even if these virtues remain slightly debated (Hemilä and Kaprio, 2009, 2011).

Instead of focusing on ROS buffering strategies, other studies tried to act more specifically on enzymatic sources of ROS. One of them is the pharmacological inhibition of Xantine Oxidase, by allopurinol. First, in both rat and mouse models of dilated cardiomyopathy, administration of allopurinol is able to decrease the level of ROS, which contributes to slow down the disease (Engberding et al., 2004; Wang Z. et al., 2016). Interestingly, in a clinical study led on HF patients, allopurinol was able to restore M-CK activity, hence to sustain the transfer of energy from mitochondria to sarcomere (Hirsch et al., 2012). More recent study revealed an improvement of walk effort resistance in patients suffering from left ventricular dysfunction, even with higher administrated dose (300 mg daily for 1 week then 600 mg daily for 3 months) (Ansari-Ramandi et al., 2017). However, in patients undergoing percutaneous intervention, allopurinol pretreatment revealed as not effective enough to modify cardiac biomarkers, and especially the level of creatine kinase or troponin-T (Alemzadeh-Ansari et al., 2017; Ansari-Ramandi et al., 2017). This last work may lead to suggest the idea that relevance of such a treatment may depend on numerous parameters.

More recently, ubiquinone (coQ10), another natural antioxidant, has received attention, because it takes part in the redox balance through the mitochondrial respiratory chain, hence playing a critical role in OXPHOS. In addition, it has been demonstrated that HF severity is proportional to the depletion of coQ10, suggesting that a supply of this antioxidant could really help in slowing down the fatal evolution of the disease (Belardinelli et al., 2005). Consequently, many clinical trials have been performed, with large patient cohorts. However, although promising, comparison of the results was challenging due to cohort heterogeneity and lack of randomization. Finally from 41 relevant studies, the most recent meta-analysis identified only 13 accurate studies (Fotino et al., 2013). From this amount of data, the beneficial effect of coQ10 on ejection fraction was validated, but the impact on long-term survival rate following the treatment remained unclear (Fotino et al., 2013). A more recent randomized study including 420 HF patients for 2 years shed new light concerning the coQ10 therapeutic strategy (Mortensen et al., 2014). Called, Q-Symbio, this large-scale study has strongly established a reduction of mortality due to cardiovascular causes as well as a decrease of hospital admission for HF among patients treated with coQ10 (Mortensen et al., 2014).

Finally, antioxidant strategy has carried out promising clinical trials, but its whole efficiency remains mixed. These drugs might mainly act as “redox sponges,” favoring ROS scavenging in a certain extent, without addressing the pathogenesis. This makes sense since deleterious oxidative stress is both a cause and a consequence in cardiovascular diseases, especially in HF. Consequently, therapeutic use of antioxidants would be a good complementary to any other strategy.

By contrast, a growing number of clues suggest that restoring the supply of “cardiac fuel” can bring sustainable solutions (Fukushima et al., 2015; Salzman et al., 2017).

### Facilitate FA uptake and oxidation

One approach involves refueling the heart, else by the stabilization or by the increase of FA uptake and/or FAO. However, this strategy seems to be dangerously close to maladaptive processes, regarding the level of dioxygen available that may not be matched. Consequently, there have been some ambiguous and contradictory data, resulting from this approach. For instance, high fat diet feeding to mice worsens TAC-induced left ventricular remodeling and this is associated with insulin resistance (Raher et al., 2008). By contrast, in hypertensive rat model, the same high fat diet treatment seems to be beneficial, as it is associated with reduction of the hypertrophic remodeling process, mostly by improving cardiac contractility (Okere et al., 2005). Equivalent results were obtained after lipid administration on the hearts of arctic ground squirrels, protecting them against adverse effects of ischemia/reperfusion (Salzman et al., 2017). All these results match with several puzzling observations of human patients (usually called the “obesity paradox”). Indeed, the risk for developing cardiovascular disease is higher in obese people, but the survival rate is also higher in these overweight patients compared to normal or low-weight patients (Oreopoulos et al., 2008; Clark et al., 2011; Nagarajan et al., 2016). By contrast, clinical trials demonstrated that polyunsaturated fatty acids administration (PUFAs) are beneficial for preventing or delaying the progression of HF, whereas monounsaturated FA supply in patients suffering from end-stage HF trigger the decline of cardiac function (Mozaffarian et al., 2005; Xin et al., 2012; Imamura et al., 2013; Masson et al., 2013).

### Decrease FA uptake and oxidation

As a mirror effect, a preferred alternative consists in the reduction of the FA uptake. This approach may avoid a toggling of the metabolic machinery, hence matching better with oxygen availability. Moreover, in accordance with the Randle cycle, a decrease of FA uptake and oxidation would be able to increase proportionally glucose use and breakdown.

As a key checkpoint in mitochondrial FA uptake, CPT1 represents a prime target in order to reduce FAO, thus, several CPT1 inhibitors have been designed. Among them, etomoxir and perhexeline received previously closer attention. Several studies in both human patients and rodent models validated some beneficial effects, and particularly their anti-ischemic virtues, by favoring the switch to glucose oxidation (Fillmore and Lopaschuk, 2013). A recent study focused on Mitochondrial Trifunctional Protein Deficiency (MTFD), a genetic disease of which the most severe neonatal form leads to cardiomyopathy and hepatic dysfunction. In this study, etomoxir-treated MTFD fibroblasts exhibited a clearing of cytotoxic fatty-acyls carnitine and improved mitochondrial respiration rate and ATP production (Lefort et al., 2017). Due to its ability to boost glucose utilization, etomoxir was previously used as an anti-diabetic agent. In addition, a 3-month treatment, led in a small group of patients, demonstrated an improvement of LV ejection fraction and exercise tolerance as well as a better overall medical condition (Schmidt-Schweda and Holubarsch, 2000). Beyond cardiac metabolism, the treatment with etomoxir can improve Ca^2+^ handling by increasing SERCA2a expression (Zarain-Herzberg et al., 1996; Rupp and Vetter, 2000). However, a larger-scale study (Etomoxir for the Recovery of Glucose Oxidation, ERGO) had been hastily stopped because HF patients rapidly exhibited a disturbing increase of transaminase blood level, suggesting a severe hepatotoxicity (Holubarsch et al., 2007). Thus, the irreversible impact of etomoxir on the hepatic tissue seems to supersede its potential beneficial effects on cardiac function.

New challenging approaches carry on more specifically targeting CPT1B, the more cardiac specific isoform. In such a purpose, oxfenicine (Sepa-Kishi et al., 2016) or perhexeline (ex anti-angina agent from the 1970's) (Chong et al., 2016) hold promising data both *in vitro* and in animal studies.

While high doses of these agents were associated with neurotoxicity and hepatotoxicity (Shah et al., 1982), more interestingly, clinical trials demonstrated that a lower dose of perhexeline plasma level specifically inhibits cardiac CPT1B (Kennedy et al., 1996). Although perhexiline virtues on LV function were demonstrated decades ago in an *ex vivo* canine model (Ono et al., 1982), there is a recent growth of interest for this metabolic agent (Chong et al., 2016). Notably, it has been demonstrated that mice fed with perhexiline preserve partly the cardiac function, reducing FAO and increasing glucose use (Yin et al., 2013). Several clinical trials in patients also validate a positive impact in electrophysiological parameters. However, recent studies trend to demonstrate that this metabolic agent also exhibits many beneficial side effects on other main effectors involved in maladaptive cardiac remodeling, such as NOX2 complex (Liberts et al., 2007; Gatto et al., 2013), Kruppel-like factor 14 (Guo et al., 2015) and mTORC1 (Balgi et al., 2009), thus, reducing inflammation, lipid metabolism and autophagy.

In addition, as a natural feedback inhibitor of CPT1, MalonylCoA can be considered as another way of slowing down FAO. Usually, this TCA byproduct is converted to AcetylCoA, and this reaction is catalyzed by MalonylCoA Decarboxylase (MCD) (Hamilton and Saggerson, 2000). Several recent data trend to validate MCD inhibition as promising therapeutic strategy. First, in a swine model of HF, administration of MCD inhibitors improves cardiac function and upregulates glycolysis (Dyck et al., 2004). More recently, MCD gene silencing in both mouse and rat are associated with a better-preserved ejection fraction following surgical induction of myocardial infarction (Masoud et al., 2014; Wu et al., 2014). In such a situation, it has been demonstrated that the preservation of cardiac function is coupled with an upholding of ATP production. However, since the experiments that yielded this information are particularly recent, this strategy has not been tried yet on HF patients.

As the terminal enzyme of the β-oxidation cycle, 3-KetoAcylCoAThiolase represents another interesting therapeutic target. Its catalytic activity can be partly inhibited by the trimetazidine. Usually used as an anti-angina treatment in more than 90 countries through the world, trimetazidine is efficient in decreasing FA oxidation, thus increasing glucose oxidation. This drug holds beneficial impact on myocardial infarction and HF (D'hahan et al., 1997; Belardinelli, 2000; Saeedi et al., 2005; Belardinelli et al., 2008; Marazzi et al., 2009; Zhou and Chen, 2014). Trimetazidine is also associated with cardioprotective effects in pressure-overload cardiac hypertrophy, where FA oxidation is already disrupted. In fact, data suggest that trimetazidine modulates the production of H+ and regulates the Ca^2+^ handling (by acting on SERCA catalytic activity), thus preserving ionic exchange and homeostasis inside the cardiac cells, which contributes to an improvement of the cardiac function (Meng et al., 2006). Ranolazine and Dichloroacetate are two other anti-angina agents which act on cardiac metabolism by increasing PDH activity, thus removing the FA-associated negative feedback upon glucose oxidation (Scirica and Morrow, 2007; Scirica et al., 2007; Gutierrez et al., 2015). Research and clinical trials demonstrated that their beneficial effects are quite equivalent to trimetazidine (Clarke et al., 1993; Gralinski et al., 1996; Battiprolu and Rodnick, 2014; Caminiti et al., 2016; Coppini et al., 2017).

### Modulate AMPK signaling pathway

In cardiac metabolism, the AMP-activated protein kinase (AMPK) acts as a “fuel gauge” due to its sensitivity to AMP/ATP and creatine/phosphocreatine ratios (Hardie, 2004; Shirwany and Zou, 2014). AMPK mediates cell catabolism and ATP production by different strategies. First, AMPK positively impacts on GLUT1 expression, GLUT4 endocytosis, and phosphofructose kinase 2 activity, thus promoting glucose uptake (Zhao et al., 2017). AMPK can also increase FAO by decreasing acetylCoA carboxylases activity, which consequently decreases the level of malonyl CoA and thus release the CPT1 inhibition. In addition, AMPK can inhibit mTORC1, which reduces protein synthesis and favors autophagy. Meanwhile AMPK reversely activates FoxOs, which contributes to glycolysis by regulating PGC1-α and Glucose-6-Phosphatase thus boosting both FAO and glycolysis (Jäger et al., 2007; Zhu et al., 2010; Kousteni, 2012; Eijkelenboom and Burgering, 2013). Notably, AMPK pathways are also involved in oxidative stress; AMPK/PKC-2α can regulate NOX2 activity (Balteau et al., 2014), while AMPK/FOXO3 activates Thrx2 in endothelial cells (Hou et al., 2010). Lastly, AMPK works in a reverse crosstalk with AKT signaling that usually favors anabolism and cell survival (Zhao et al., 2017). Hence, AMPK activation is foreseen as an up-and-coming therapeutic strategy (Beauloye et al., 2011). Among them, metformin, a common drug for the treatment of Type 2 diabetes, has caught careful attention. Indeed, metformin induced-AMPK activation can protect from myocardial injury and cell apoptosis following ischemia (Kim et al., 2011; Yin et al., 2011) and it improves cardiac function (and oxidative metabolism) in rodent models of HF (Benes et al., 2011; Fu et al., 2011). Yet, long-term activation of AMPK is deleterious for cardiac function, due to negative feedback on CPT1 expression and activity, thus reducing FA use and metabolism. Cardioprotective effects of metformin were also demonstrated in rat model of chronic HF (Wang et al., 2011). However, a recent meta-analysis validates with caution this beneficial impact only on experimental myocardial infarction (Hesen et al., 2017). And this is the line with observations led in clinical trials. In fact, in non-diabetic patients who underwent acute myocardial infarction, clinical studies about metformin treatment remain disappointing or contradictory (Lexis et al., 2014). Moreover, although beneficial effects were suggested in a clinical trial focusing in HF with preserved ejection fraction (Shah et al., 2010), metformin seems to exacerbate cardiac disruption in end-stage HF by increasing lactate acidosis (Wong et al., 2009; Hou et al., 2010; Doenst et al., 2013). This data validates the “Janus double-headed” role of AMPK in cardiac metabolism. Thus, although interesting, AMPK treatment seems to be case-by-case-dependent. Consequently, caution remains needed concerning the use of AMPK activators in clinical trials.

### Increase blood glucose level and release

In cardiac disease, insulin tolerance or resistance is a crucial parameter. Upstream insulin and glycolysis, glucagon-like peptide-1 (GLP-1) is a gut-secreted incretin (metabolic hormone) which can promote the release of insulin after meals to activate cell glucose uptake from blood (Nadkarni et al., 2014). In experiments using dogs and rats, GLP-1 infusion can increase glucose uptake and improve cardiac function (Nikolaidis et al., 2004a; Sokos et al., 2006; Poornima et al., 2008). Regarding its short half-life in plasma, pharmacological agonists of GLP-1 have been developed with promising but sometimes contradictory results (Liu et al., 2014; Margulies et al., 2014; Trujillo et al., 2015). GLP-1 receptor blockage abolished IPC cardioprotective effects in a rat model of myocardial infarction, suggesting a deep impact of GLP-1 on pathways involved in IPC (Basalay et al., 2016). Then, recent studies in H9c2 myoblasts demonstrated, in particular, that GLP-1 and analogs can decrease oxidative stress and also hypoxia/reoxygenation-induced apoptosis via activation of PI3K/AKT pathway (Chang et al., 2014; Jiang et al., 2016; Petersen et al., 2016). Another approach demonstrated that GLP-1 can also activate AMPK, which reduces hyperglycemia-induced NOX2 activity in adult rat cardiomyocytes (Balteau et al., 2014). Beyond glycemic control, GLP-1 and analogs exhibit side anti-inflammatory and anti-atherogenic effects (Anagnostis et al., 2011; Petersen et al., 2016). Such a pleiotropic action has thus very recently led them to greater and promising interest from bench to bedside.

### Myocardial stimulation by catecholamines

Last but not least, neurotransmitters can be viewed as other way of positively impacting in cardiac metabolism (Slatton and Eichhorn, 1996; Bouzamondo et al., 2001; Lechat, 2004). Indeed, sympathetic and parasympathetic stimulations by catecholamines are crucial in cardiac adaptive processes. As previously described, metabolic demands increase during rigorous exercise. It is well described that short-term β-adrenergic stimulation increases glucose uptake and oxidation (Morisco et al., 2005; Nagoshi et al., 2011). Stimulation by the use of an agonist (epinephrine) can also favor Ca^2+^ handling cycle, with potential beneficial in myocardial contractility (Collins-Nakai et al., 1994; Briston et al., 2014; Grimm et al., 2015; Ho et al., 2016). Moreover, recent study demonstrated that a selective β3-adrenergic receptor agonist (BRL_37344_) favors left ventricular relaxation in Langendorff-perfused rat hearts via the NO/cGMP/PKG axis, thus by activating specific redox signaling and favoring beneficial adaptive process (Angelone et al., 2008; Cannavo and Koch, 2017). NMR spectroscopy in rat heart also demonstrated that acute β-adrenergic receptor stimulation by isoprotenerol leads to a sudden lactate production from glycogen stock (Khemtong et al., 2015). However, long-term activation leads to insulin resistance, making therapy challenging (Morisco et al., 2005; Ciccarelli et al., 2011). In fact, β-adrenergic receptor stimulation overactivates cAMP/PKA signaling pathway, leading to a decrease in insulin-dependent GLUT4 membrane translocation and expression, and thereby to a disruption of glucose uptake (Mangmool et al., 2016).

By contrast, long-term blockade with a non-selective β-adrenergic and α1-adrenergic antagonist (carvedilol) reduces FA oxidation by favoring glucose use and breakdown in HF patients, which may provide longer-term and more efficient therapeutic benefits (Eichhorn et al., 1990; Eichhorn, 1998, 2000). In fact, more recent studies demonstrated that carvedilol encloses a bunch of beneficial side-effects including antioxidant, anti-inflammatory and anti-apoptotic virtues, notably by favoring ROS scavenging systems and by activating specific miRNA involved in cardiomyocyte survival (miR-125b-5, miR-199-3p, miR-214) (Bayoumi et al., 2017, p. 125; Park et al., 2016). Network meta-analyses remain ambivalent concerning carvedilol superiority on mortality benefits, when compared with specific β1-adrenergic receptor blockers, but several bias (sampling, dose administration) made these studies to be taken with caution (Kveiborg et al., 2007; DiNicolantonio et al., 2013; Zhang et al., 2016; Li J. et al., 2017).

Although non-exhaustive, these studies underline an updated interest for β-blockers. Combined administration of carvedilol with antioxidant could be an interesting therapeutic strategy in cardiac diseases, and first clinical trials have shown encouraging potential (Budni et al., 2013; El-Shitany and El-Desoky, 2016).

## Conclusions

Cardiac metabolism works in a finely tuned fashion: with enough fuel and dioxygen, cardiomyocytes can rapidly and efficiently generate ATP to convert into mechanical force. The current body of research now clearly demonstrates that maladaptive metabolic behaviors as well as ROS/RNS double-edged effects are intimately involved in both the genesis as well as the progression of cardiac diseases. As a result, though many therapies have been developed for years, none of them can be considered as a panacea, since the fact to reconcile fuel sources and oxidative capacity is the toughest challenge. For now, the best therapeutic approach may be a combination of drugs with complementary virtues. On the bedside of patients, a case-by-case approach is therefore necessary and based on specific patient clinical history.

## Author contributions

AA, XP, and LX wrote the manuscript. AA prepared the figures.

### Conflict of interest statement

The authors declare that the research was conducted in the absence of any commercial or financial relationships that could be construed as a potential conflict of interest.

## Abbreviations

AMPK: AMP-activated protein kinase
CK: Creatine Kinase
CRATs: Carnitine O-acetyl transferases
COTs: Carnitine octanoyltransferases
CPT1: Carnitine palmitoyl transferase 1
DCM: Dilated cardiomyopathy
FA: Fatty acids
FAO: Fatty acids β-oxidation
FACS: Fatty acyl CoA Synthase
G6P: Glucose 6-Phosphate
GLP-1: Glucagon-like peptide-1
GLUT: Glucose transporter
HF: Heart failure
HIF1α: Hypoxia-inducible factor 1α
IPC: Ischemic preconditioning
JNK: (Stress-activated kinase)/c-jun N-terminal kinase
LDH: Lactate dehydrogenase
MAPK: Mitogen-associated protein kinase
PDH: Pyruvate dehydrogenase
PDK1: Pyruvate deshydrogenase kinase 1
PGC-1α: Peroxisome proliferator-activated receptor γ coactivator-1α
PPAR: Peroxisome proliferator-activated Receptor
ROS: Reactive oxygen species
RXR: Retinoid X receptor
SERCA: Sarcoplasmic reticulum Ca^2+^ ATPase
TAC: Transversal aortic constriction
TCA cycle: Tricarboxylic cycle.
